# Design, Expression, Purification, and Characterization of a YFP-Tagged 2019-nCoV Spike Receptor-Binding Domain Construct

**DOI:** 10.3389/fbioe.2020.618615

**Published:** 2020-12-21

**Authors:** Tobias Bierig, Gabriella Collu, Alain Blanc, Emiliya Poghosyan, Roger M. Benoit

**Affiliations:** ^1^Laboratory of Nanoscale Biology, Division of Biology and Chemistry, Paul Scherrer Institute, Villigen, Switzerland; ^2^Department of Biology, ETH Zürich, Zurich, Switzerland; ^3^Center for Radiopharmaceutical Sciences ETH-PSI-USZ, Paul Scherrer Institute, Villigen, Switzerland

**Keywords:** fluorescent spike RBD fusion protein, YFP-spike_RBD, protein reagent for COVID-19 research, 2019-nCoV spike-RBD production using standard cell culture equipment and techniques, secreted glycosylated YFP-labeled protein, mammalian protein secretion

## Abstract

2019-nCoV is the causative agent of the serious, still ongoing, worldwide coronavirus disease (COVID-19) pandemic. High quality recombinant virus proteins are required for research related to the development of vaccines and improved assays, and to the general understanding of virus action. The receptor-binding domain (RBD) of the 2019-nCoV spike (S) protein contains disulfide bonds and N-linked glycosylations, therefore, it is typically produced by secretion. Here, we describe a construct and protocol for the expression and purification of yellow fluorescent protein (YFP) labeled 2019-nCoV spike RBD. The fusion protein, in the vector pcDNA 4/TO, comprises an N-terminal interferon alpha 2 (IFNα2) signal peptide, an eYFP, a FLAG-tag, a human rhinovirus 3C protease (HRV3C) cleavage site, the RBD of the 2019-nCoV spike protein and a C-terminal 8x His-tag. We stably transfected HEK 293 cells. Following expansion of the cells, the fusion protein was secreted from adherent cells into serum-free medium. Ni-NTA immobilized metal ion affinity chromatography (IMAC) purification resulted in very high protein purity, based on analysis by SDS-PAGE. The fusion protein was soluble and monodisperse, as confirmed by size-exclusion chromatography (SEC) and negative staining electron microscopy. Deglycosylation experiments confirmed the presence of N-linked glycosylations in the secreted protein. Complex formation with the peptidase domain of human angiotensin-converting enzyme 2 (ACE2), the receptor for the 2019-nCoV spike RBD, was confirmed by SEC, both for the YFP-fused spike RBD and for spike RBD alone, after removal of YFP by proteolytic cleavage. Possible applications for the fusion protein include binding studies on cells or *in vitro*, fluorescent labeling of potential virus-binding sites on cells, the use as an antigen for immunization studies or as a tool for the development of novel virus- or antibody-detection assays.

## Introduction

**FIGURE 1:**
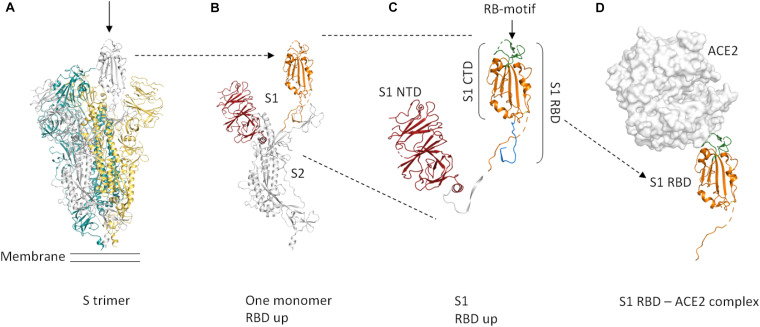
Cartoon representation of the protein domains of the 2019-nCoV spike protein. Dashed lines in the structure indicate flexible regions that are not defined. Glycosylations are not shown. The domain boundaries are based on UniProt entry P0DTC2. **(A)** The S protein forms a trimer that is exposed on the virus surface. The monomers forming the trimer are shown in gray, yellow, and teal. In the prefusion conformation shown here (PDB entry 6VSB, Wrapp et al., 2020), only one of the three RBD domains is rotated up (chain shown in gray, arrow) in a conformation that is accessible for ACE2 binding. **(B)** Only the chain shown in gray in **(A)** is depicted here. A selection of important features is shown in color (orange *=* S RBD amino acids 319–541, red *=* S1 NTD. **(C)** Close-up view of S1, showing the S1 NTD (amino acids 13–303, red), S1 CTD (amino acids 334–527, orange and green), S_RBD as in our construct (amino acids 319–527, shown in orange and green) and the receptor-binding motif (amino acids 437–508, green). The linker that connects S RBD and S1 NTD is shown in white. The stretch shown in blue comprises amino acids 528–541. These most C-terminal residues of S RBD are not included in our construct. **(D)** ACE2 binding to S RBD. The ACE2 peptidase domain is shown as a surface representation (white). S RBD is shown in orange and green. The green region indicates the receptor-binding motif. For this Figure, the S RBD of PDB entry 6M17 (Yan et al., 2020) was superimposed onto the S RBD of PDB entry 6VSB (Wrapp et al., 2020). The S RBD of PDB entry 6VSB and the ACE2 peptidase domain of PDB entry 6M17 are shown. The Figure was created using PyMOL (The PyMOL Molecular Graphics System, Version 2.3.4 Schrödinger, LLC).

The membrane-anchored, trimeric spike (S) glycoproteins are the most prominent protrusions on the surface of the novel coronavirus (2019-nCoV) (Figure 1A). Coronavirus (CoV) spike proteins typically comprise two subunits (Figure 1B). The S1 subunit is responsible for receptor binding and the S2 subunit is involved in fusing the membranes of the virus and the host (Li, 2016). The S1 subunit is composed of an N-terminal domain (S1-NTD) and a C-terminal domain (S1-CTD) (Figure 1C; Li, 2016). S1-CTD comprises two sub-domains, one functioning as a core structure, the other one as a receptor-binding motif (Figure 1C; Li, 2016). The receptor-binding domain of 2019-nCoV binds human angiotensin-converting enzyme 2 (ACE2) with high affinity (Wrapp et al., 2020). The overall ACE2—S1-RBD binding mode is depicted in Figure 1D.

The spike RBD is an important target for drug discovery research (Toelzer et al., 2020) and for the development of vaccines (Wang et al., 2020; Wrapp et al., 2020). Within the S trimer, the receptor-binding domains (RBDs) can be in a down conformation or alternatively in an up conformation, the latter being the receptor-accessible state (Figure 1A; Wrapp et al., 2020). Recent complex structures (Wang et al., 2020; Yan et al., 2020) confirmed that a single spike RBD, taken out of the trimeric context, is capable of binding its human receptor ACE2 (Figure 1D).

S-RBD contains disulfide bonds and is glycosylated (Wrapp et al., 2020; Yan et al., 2020). To obtain the correct co-translational modifications, the protein domain is therefore typically produced by secretion using eukaryotic cells. For example, Wang et al. secreted an S-RBD construct (amino acids 319–541) with a C-terminal monomeric constant domain (Fc) of immunoglobulin G (IgG) fusion using HEK293T cells and transient transfection. The RBD construct of Wrapp et al. (amino acids 319–591) also contained a C-terminal monomeric Fc tag. They secreted the protein from transiently transfected FreeStyle293F cells. Yan et al. used a C-terminally mFc tagged RBD construct (amino acids 319–541) from a commercial source (Sino Biological Inc.).

A wide range of fusion proteins are available for cytoplasmic protein overexpression, however, only few fusion proteins are applicable to protein secretion (Dalton and Barton, 2014). The Fc domain of IgG and human serum albumin are the only fusion proteins that are routinely used in the context of secreted proteins (Dalton and Barton, 2014). The Fc domain of IgG is frequently used as a C-terminal fusion protein for S-RBD production. Our aim was to instead express S-RBD fused to a fluorescent protein.

Eukaryotic protein expression and secretion can be carried out in a variety of formats, including adherent cell cultures in flasks (e.g., Macdonald et al., 2006; Dalton and Barton, 2014) or suspension cell cultures (reviewed in Dalton and Barton, 2014). Furthermore, expression can be performed by large scale transient transfection (e.g., Wang et al., 2020; Wrapp et al., 2020) over a short time frame, or over an extended expression period using stably transfected cells (Chaudhary et al., 2012). Our aim was to implement a protocol that can be carried out using standard cell culture equipment and hence can be widely adapted. The combination of stably transfected cells and adherent cell cultures in flasks allows continuous production of protein in standard CO_2_ incubators, without depending on CO_2_ shaking incubators.

Here, we describe a construct and protocol for the production and purification of milligram amounts of N-terminally YFP-labeled spike RBD. The domain boundaries of our receptor-binding domain (RBD) construct are based on the construct used for the crystal structure by Wang et al. (Cell 2020, PDB entry 6LZG), comprising amino acids 319–527, which also includes the receptor-binding motif (amino acids 437–508, UniProtKB—P0DTC2) (Figures 1C,D).

Expression is performed by secretion into serum-free medium from adherent, stably transfected HEK293 cells. The protocol involves only standard cell culture techniques and equipment. Our experiments confirmed that the fusion protein (also after proteolytic removal of YFP) binds to the human ACE2 peptidase domain.

## Materials and Methods

### Plasmids

The DNA coding for the IFNα2-eYFP-FLAGtag-PreScission_site-S_RBD-8xHis-tag-StopStop fusion protein was ordered from Genewiz, cloned into the *Hin*dIII and *Xba*I sites of pcDNA 4/TO (Invitrogen). The human ACE2 peptidase domain (amino acids 19–615) construct with an N-terminal interleukin-2 (IL-2) peptide and a C-terminal PreScission site and an 8xHis-tag and two stop codons was ordered as a FragmentGENE from Genewiz and cloned into the *Kpn*I and *Not*I sites of pcDNA 4/TO.

### Transfection

#### YFP-S_RBD

Adherent HEK293 cells were grown to ∼90% confluence in a 9 cm diameter cell culture dish at 37°C, 5% CO_2_. Just before transfection, the cells were washed with 10 ml PBS. Twenty two micrograms plasmid DNA and 50 μg of 25 kDa, linear polyethylenimine (PEI) were mixed in a sterile 15 ml Falcon tube and incubated at room temperature for 10 min with occasional gentle mixing. Next, 5 ml of Dulbecco’s Modified Eagle Medium (DMEM), with high glucose and L-glutamine (Bioconcept), without FBS, were added to the DNA-PEI mixture, followed by another 10 min incubation at room temperature with occasional mixing. Thereafter, the PBS was removed from the cells and the transfection mixture was added onto the cells and distributed well. After incubation at 37°C, 5% CO_2_ for 6 h, 10 ml of DMEM high glucose supplemented with 1% FBS were added, and the cells were incubated at 37°C, 5% CO_2_ overnight. The next day, the cells were split 1:10 and grown in DMEM high glucose medium supplemented with 10% FBS.

#### ACE2 Peptidase Domain

The transfection procedure for the ACE2 construct was identical to the procedure described for YFP-S_RBD, except that only 11.5 μg of plasmid DNA were used and that the confluence of the HEK293 cells was ∼50%. Furthermore, the 10 ml of DMEM high glucose supplemented with 1% FBS were added 1.5 h after addition of the transfection mixture to the cells.

### Selection of Stable Cell Lines

#### YFP-S_RBD

After another overnight incubation, the medium was replaced by fresh medium of the same composition, and supplemented with Zeocin (InvivoGen) to a final concentration of 100 μg/ml and Penicillin-Streptomycin (PAN Biotech) to a final concentration of 100 U/ml. The selective medium was exchanged every 2–3 days until only Zeocin-resistant cells remained and the cells were confluent. Nine days after transfection, the cells were trypsinized and transferred to a 75 cm^2^ cell culture flask in 20 ml selective medium. Fourteen days after transfection, 3/5 of the cells in the 75 cm^2^ flask (∼25% confluent) were split into a new 75 cm^2^ flask for expression, while the other 2/5 were transferred to another flask as a backup and for freezing.

#### ACE2 Peptidase Domain

One day after transfection, the cells (now confluent) were split 1:10 and grown in DMEM high glucose medium supplemented with 10% FBS. After another overnight incubation, the medium was replaced by fresh medium of the same composition, and supplemented with Zeocin (InvivoGen) to a final concentration of 100 μg/ml and Penicillin-Streptomycin (PAN Biotech) to a final concentration of 100 U/ml. The selective medium was exchanged every 2–3 days until only Zeocin-resistant cells remained and the cells were confluent. Twelve days after transfection, the cells (∼90% confluent) were trypsinized and transferred to a 75 cm^2^ cell culture flask in 20 ml selective medium. Sixteen days after transfection, the cells were confluent.

### Protein Expression

#### YFP-S_RBD

Sixteen days after transfection, when the cells in the expression flask were ∼50% confluent, they were washed twice with PBS, and 15 ml of serum-free, selective expression medium (Opti-MEM I reduced serum medium, Gibco REF 11058-021) supplemented with 100 μg/ml Zeocin (InvivoGen cat no ant-zn) and 3 μg/ml tetracycline) were added. The selective expression medium was collected every 2–3 days and replaced with fresh medium of the same composition. Once in the serum-free medium, the cells reached confluence within 10 days. The supernatant medium collected from the confluent culture typically contained some detached cells, which were removed by centrifugation at room temperature, 1,500 rcf for 10 min.

For upscaling, the cells were expanded into two cell culture flasks with 150 cm^2^ surface area each. The cells were grown to confluence and then washed twice in PBS before adding serum-free medium for expression.

#### ACE2 Peptidase Domain

Sixteen days after transfection, the HEK293 cells expressing ACE2 peptidase domain were confluent in a 75 cm^2^ area cell culture flask. The cells were washed with PBS twice and serum-free medium (Opti-MEM I reduced serum medium, Gibco REF 11058-021) supplemented with 100 μg/ml Zeocin (InvivoGen cat no ant-zn) and 3 μg/ml tetracycline) was added for expression.

### Protein Purification

#### Ni-NTA IMAC

##### YFP-S_RBD

After removal of detached cells by centrifugation, the expression medium containing the secreted fusion protein was supplemented with 1/4 tablet of protease inhibitor (complete EDTA-free protease inhibitor cocktail tablets, Roche Diagnostic GmbH, 45148300) and transferred to a 15 ml Falcon tube containing 200 μl of washed, pre-equilibrated Nickel-nitrilotriacetic acid (Ni-NTA agarose) (Qiagen Cat. No. 30210). The solution was incubated at 4°C with occasional agitation until the next batch of secreted protein became available. Then, the Ni-NTA resin was collected by centrifugation at 1,500 rcf, 10 min at 4°C. The supernatant was discarded and the fresh batch of clarified, protease inhibitor treated medium was added to the resin. This process was repeated until the Ni-NTA agarose clearly became yellow. The Ni-NTA resin was then collected by centrifugation (1,500 rcf, 10 min, 4°C), the supernatant was discarded, and the resin was resuspended in wash buffer (50 mM Tris pH 8.0, 300 mM NaCl) and transferred to a column. The resin was washed six times with 1 ml of ice-cold wash buffer per wash, using gravity flow. The protein was eluted in three 300 μl steps in 50 mM Tris pH 8.0, 300 mM NaCl, 350 mM imidazole. For the upscaled purification, 1 ml of Ni-NTA agarose was used.

##### ACE2 peptidase domain

ACE2 peptidase domain was purified using the same protocol as for YFP-S_RBD. A total of 60 ml of medium were collected over a timeframe of 12 days. Three hundred microliter of washed, pre-equilibrated Ni-NTA agarose (Qiagen Cat. No. 30210) were used. The protein was eluted in four 300 μl steps in 50 mM Tris pH 8.0, 300 mM NaCl, 350 mM imidazole. The total protein yield was 350 μg.

#### SEC

The IMAC-purified proteins were centrifuged for 10 min at 17,000 rcf, 4°C and run on a Superdex 200 Increase 10/300 GL column (Code 28-9909-44) in 50 mM Tris pH 8.0, 150 mM NaCl, at a flow rate of 0.4 ml/min on an ÄKTA Ettan system at 4°C. For binding studies, separate proteins were centrifuged 10 min at 17,000 rcf, 4°C. Equimolar amounts of the supernatants were then mixed and incubated on ice for 1 h prior to the SEC run. For the complex containing the YFP-S_RBD fusion protein, 22 μg of SEC-purified YFP-S_RBD were mixed with an equimolar amount of IMAC-purified ACE2 peptidase domain, and the volume was adjusted to 500 μl using SEC buffer. For the complex containing cleaved S_RBD without YFP, 34 μg of IMAC-purified S_RBD were mixed with an equimolar amount of IMAC-purified ACE2 peptidase domain, and the volume was adjusted to 500 μl using SEC buffer. After the incubation step, before the SEC run, the complexes were again centrifuged for 10 min at 17,000 rcf, 4°C.

### Pull-Down Assay

YFP-S_RBD and ACE2 peptidase domain protein samples in 50 mM Tris pH 8.0, 150 mM NaCl, were separately centrifuged at 17,000 rcf for 10 min at 4°C to remove aggregates. Forty micrograms of YFP-S_RBD and an 1.5-fold molar excess of ACE2 peptidase domain were mixed and incubated on ice for 1 h. In the control sample, YFP-S_RBD was omitted. The mixtures were centrifuged at 17,000 rcf for 15 min at 4°C and the supernatant solutions were mixed with 400 μl of pre-equilibrated ANTI-FLAG M2 affinity gel (Sigma-Aldrich A2220) and incubated overnight at 4°C. The FLAG resin was then washed 5x with 1.5 ml of wash buffer (50 mM Tris pH 8.0, 150 mM NaCl). Between washes, the resin was collected by centrifugation at 3,000 rcf, 4°C for 3 min. The proteins were eluted in 500 μl of wash buffer supplemented with 200 μg/ml FLAG peptide. The protein solutions were concentrated to ∼30 μl in 10 kDa cutoff centrifugal concentrators (Amicon) and analyzed by SDS-PAGE.

### Electron Microscopy

#### Negative Stain Grid Preparation

3.5–4 μl of purified protein (0.125 mg/mL) were first applied to a glow discharged, carbon coated grid (Plano, Germany), thereafter excess liquid was blotted away using filter paper and grids were stained with 1–2% uranyl acetate solution.

#### Cryo-EM Grid Preparation

The protein peak obtained from SEC was collected and concentrated to 0.6 mg/ml. Cryo-EM grids were prepared by applying 3.5 μl of protein to the glow-discharged Quantifoil R1.2/1.3 200-copper mesh grids from Electron Microscopy Science (Q2100-CR1.3). The grids were blotted for 3 s, plunge-frozen in liquid ethane using a Vitrobot Mark IV (Thermo Fischer Scientific), operated at 4°C and 100% humidity, and stored in liquid nitrogen until cryo-EM data collection.

#### EM Data Acquisition

Data acquisition was performed using a JEM2200FS transmission electron microscope (JEOL, Tokyo, Japan) equipped with an in-column energy filter and a field emission gun. Micrographs were recorded with K2/XP direct electron detector (Gatan, Ametek) and GMS3 software (Gatan, Ametek).

### Deglycosylation

For analysis by SDS-PAGE, 5 μl 1x PBS and 2 μl (1 U) PNGase F (from *Elizabethkingia meningoseptica*, expressed in *E. coli*, Sigma Aldrich F8435-50UN) were added to 40 μl of SEC purified YFP-S_RBD (0.24 mg/ml protein concentration), followed by overnight incubation at room temperature. For mass spectrometry, the protein deglycolysation was achieved by incubating 50 μl (0.24mg/ml) protein solution with 1 μl of PNgase F (500U, glycerol free) at 37°C overnight.

### Mass Spectrometry

LC/MS analysis was performed on a waters LCT Premier mass spectrometer (ESI-TOF) and HPLC Waters 2795. Samples were chromatographed on a Reprosil-PUR 2000 C18-AQ column (3 μm, 100 × 2 mm) heated to 50°C using the conditions shown in Table 1:

**TABLE 1:** HPLC Method description.

Time	Solvent A	Solvent B	Flow
0 min	2%	98%	0.5 ml/min
2 min	25%	75%	0.5 ml/min
25 min	50%	50%	0.5 ml/min
30 min	80%	20%	0.5 ml/min

*Solvent A: acetonitrile with 0.1% formic acid, Solvent B: water with 0.1% formic acid.*

### SDS-PAGE Analysis

For protein analysis by sodium dodecyl sulphate-polyacrylamide gel electrophoresis (SDS-PAGE), NuPAGE 4-12% BisTris, 1.0 mm × 12 well (invitrogen by Thermo Fisher Scientific, NP0322BOX) gels were run in NuPAGE MES SDS running buffer (Invitrogen NP0002) at 150 V. NuPAGE LDS Sample Buffer (Invitrogen) was used with or without DTT. PageRuler Plus Prestained Protein Ladder (Thermo Scientific 26619) was used. Proteins eluted from analytical SEC were concentrated in 10 kDa cutoff spin concentrators prior to analysis by SDS-PAGE. Staining was performed using InstantBlue Coomassie Protein Stain (Expedeon, ISB1L).

## Results

### Construct Design

Our aim was to produce high-quality, soluble 2019-nCoV spike RBD labeled with a fluorescent protein for easy detection. Spike RBD contains disulfide bonds and N-glycosylations (see e.g., PDB entry 6M17, Yan et al., 2020; PDB entry 6VSB, Wrapp et al., 2020; PDB entry 6LZG, Wang et al., 2020). Therefore, this protein domain is usually produced by secretion from eukaryotic cells.

Only few fusion proteins are commonly used for secreted proteins, notably the constant domain (Fc) of IgG and human serum albumin (Dalton and Barton, 2014). We instead used yellow fluorescent protein as a fusion protein. Analysis of enhanced yellow fluorescent protein (eYFP, Ormö et al., 1996), using the NetNGlyc 1.0 server and the NetOGlyc 4.0 server (Steentoft et al., 2013), revealed no N-glycosylation sites, but a single putative O-glycosylation site, just above threshold, within the YFP sequence. Analysis of the YFP structure showed that the putative O-glycosylation site is near the surface of the protein. Furthermore, secretion of the enhanced green fluorescent protein (eGFP) has previously been described (Román et al., 2016). GFP is nearly identical to YFP in structure and sequence, and also contains the putative O-linked glycosylation site. This same publication (Román et al., 2016) also suggested improved protein secretion levels when using the interferon alpha 2 (IFNα2) signal peptide, compared to a number of commonly used signal peptides, including the signal peptide of interleukin-2 (IL-2). For this reason, we used the IFNα2 signal peptide in our construct. As in the construct described by Román et al. (2016), we also placed the signal peptide directly upstream of the fluorescent protein, however, we left out the start methionine of YFP, since translation starts at the start ATG of the signal peptide upstream of the YFP. We inserted a short linker (translating into Gly-Ser) between the signal peptide and YFP, which allowed the insertion of a *Bam*HI restriction endonuclease recognition sequence for later use of the vector with the signal peptide for other targets.

The construct was designed for insertion into the *Hin*dIII and *Xba*I sites of the vector pcDNA 4/TO (Invitrogen), a mammalian expression vector that allows tetracycline-inducible expression from a CMV promoter in cells expressing the tetracycline repressor protein, and constitutive expression in cells not containing the tetracycline repressor protein. At the 5’ end of the insert, we entered a *Not*I site containing a partial Kozak sequence (GCGGCCGCCATGG). To obtain a complete, optimal Kozak sequence, we included an additional nucleotide between the *Not*I site and the start codon. The penultimate residue (first amino acid after the start codon) in the signal peptide is alanine, resulting in an optimal ATGG DNA sequence (Kozak, 1987). A FLAG-tag for detection of the fusion protein or cleaved-off YFP was included at the C-terminus of YFP upstream of a human rhinovirus 3C protease cleavage site. The FLAG-tag also served as an additional purifications tag, for example for pull-down assays with multiple proteins that all contain a His-tag. The sequence coding for the Leu-Glu sequence (first two amino acids of the 3C protease recognition site) contains an *Xho*I restriction endonuclease recognition site, for later use of the vector with the signal peptide and YFP for other targets. The sequence coding for the 2019-nCoV-spike_RBD with a C-terminal, non-cleavable 8x His-tag and two stop codons, was inserted just downstream of the rhinovirus 3C protease site.

The His-tag, in particular an octahistidine tag, is frequently used and typically works well for the purification of secreted proteins (e.g., Wrapp et al., 2020).

**FIGURE 2:**
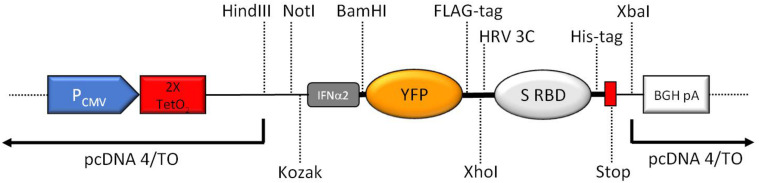
Schematic overview of the key features of the expression region of the construct (not to scale). CMV promoter (P_CMV_), tetracycline operators (2× TetO_2_), Kozak consensus sequence (Kozak), human interferon alpha 2 (IFNα2) signal peptide including start codon for the complete fusion protein, yellow fluorescent protein (YFP), human rhinovirus 3C protease cleavage site (HRV 3C), 2019-nCoV spike RBD (S RBD), 8xHis-tag (His-tag), two stop codons (Stop), BGH polyadenylation sequence (BGA pA). Important unique restriction enzyme recognition sites are indicated.

The resulting expression construct is depicted in Figure 2.

### Expression

We transfected the expression plasmid into HEK293 cells and generated stable cells by selection with Zeocin. We then expanded the adherent, stably transfected cell culture in a flask with 75 cm^2^ surface area. When a confluence of ∼50% was reached, the DMEM/FBS medium was replaced by serum-free Opti-MEM medium. We used serum-free medium for expression because serum contains proteins, such as bovine serum albumin (BSA), that are unfavorable for the subsequent purification steps and can lead to impurities in the purified protein solution.

The supernatant medium was collected three times a week, clarified by centrifugation, supplemented with protease inhibitor, and successively incubated with the same 200 μl Ni-NTA agarose batch. This process was repeated until the Ni-NTA agarose clearly turned yellowish in color. This stage was reached after nine sequential incubations, each with 12–15 ml medium.

We analyzed the YFP fluorescence of each medium batch that we collected during the initial expression. Comparison to the YFP fluorescence of a purified YFP of known concentration allowed an approximate initial estimation of the amount of secreted protein. Twelve milliliters of medium from 48 h incubation with a confluent culture with 75 cm^2^ area typically produced a fluorescence peak height of ∼900 relative fluorescence units, which corresponds to a YFP concentration of ∼2 μg/ml.

The cells, originally at ∼50% confluence, reached ∼90–100% confluence within a week in serum-free medium, and a subpopulation of cells detached in confluent cultures and had to be removed from the medium by centrifugation prior to addition to the Ni-NTA resin. After reaching confluence, the amount of protein secreted into the medium remained stable over more than 6 weeks, based on fluorescence measurements (data not shown).

To upscale protein production, we expanded the stably transfected cells from a backup plate to two larger flasks (150 cm^2^ surface area each). In the original expression flask, the cells displayed a slower growth after changing to serum-free medium, and the amount of secreted protein increased significantly as the cells reached higher confluence. Furthermore, confluent cultures remained productive for several weeks. For those reasons, we grew the larger scale cultures to confluence before changing to serum-free medium.

We then collected medium from one 75 cm^2^ flask and two 150 cm^2^ flasks and sequentially incubated the collected medium with a 1 ml batch of Ni-NTA agarose until the resin turned yellow (∼280 ml medium total, collected over 14 days).

### Protein Purification

The Ni-NTA resin was washed and the protein was eluted in an imidazole-containing buffer.

**FIGURE 3:**
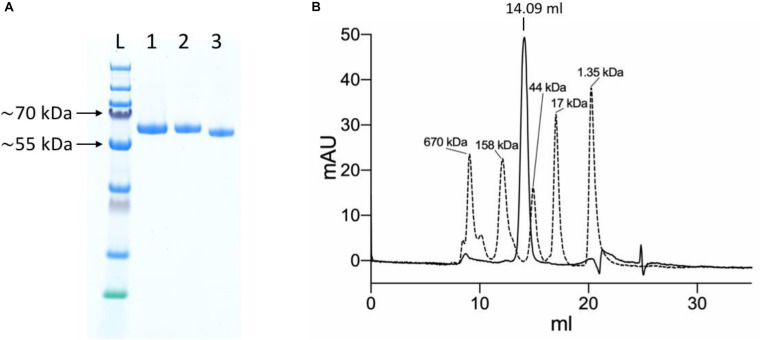
Purification of the YFP-S_RBD fusion protein. **(A)** SDS-PAGE analysis. L, Ladder; 1, YFP-S_RBD from Ni-NTA IMAC; 2, YFP-S_RBD from SEC; 3, YFP-S_RBD from SEC deglycosylated with PNGase F. **(B)** 280 nm absorbance trace from an analytical SEC run with the non-deglycosylated protein. The trace from a YFP-S_RBD SEC run (solid line) is shown overlaid with the trace of a SEC run with a standard (bio-rad gel filtration standard). Based on a calibration curve using the bovine γ-globulin (158 kDa), chicken ovalbumin (44 kDa) and horse myoglobin (17 kDa) peaks, the retention volume suggests an approximate molecular mass of ∼92 kDa. The calculated molecular weight of YFP-S_RBD, based on protein sequence, excluding glycosylations and assuming signal-peptide cleavage, is ∼54 kDa.

The initial small scale IMAC purification from 200 μl Ni-NTA resin yielded 280 μg protein of high purity (Figure 3A). The approximate amount of protein in the medium that was applied to the Ni-NTA resin, based on the fluorescence measurements, was ∼265 μg, which turned out to be a slight underestimate.

The protein solution was monodisperse according to analytical SEC, resulting in a single main SEC peak at approximately the expected retention volume (Figure 3B).

The upscaled purification from 1 ml Ni-NTA resin yielded 3.3 mg of pure protein after IMAC. The binding capacity of the Ni-NTA resin we used is up to 50 mg/ml according to supplier specifications. In both purifications, an excess of Ni-NTA resin was used. The significantly increased yield in the large-scale purification can be explained by the fact that the initial rounds of expression of the small scale expression were performed using non-confluent cultures that produced a significantly smaller amount of protein than the confluent cultures used for large scale expression. Based on the large scale expression, the yield of Ni-NTA purified protein per 100 cm^2^ of confluent culture, collected over a timeframe of 14 days and using 75 ml of medium, was 0.9 mg. The protein yield that can be expected is hence ∼9 mg per liter of medium.

### Protein Characterization

**FIGURE 4:**
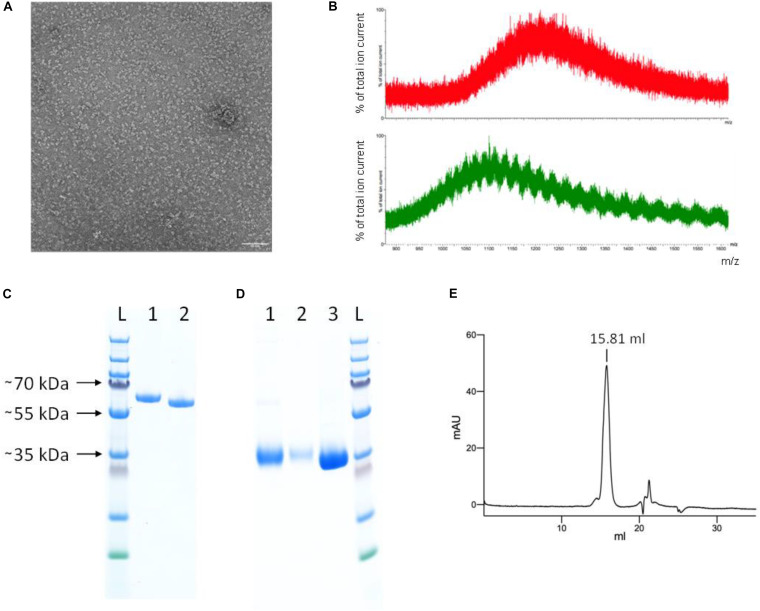
Protein characterization. **(A)** Negative staining electron micrograph of SEC-purified YFP-S_RBD fusion protein Scale bar 50 nm. **(B)** Mass spectrometry results of untreated (red, top) and PNGase F treated (green, bottom) YFP-S_RBD fusion protein. The data shows that the protein contains glycosylations. Due to glycosylation heterogeneities, a clear mass could not be determined. PNGase F treatment reduced the mass but did not remove all glycosylations, indicating that PNGase F—resistant glycosylations, such as O-linked glycosylations, are also present. **(C,D)** SDS-PAGE analysis. **(C)** YFP-S_RBD. L, Ladder; 1, Reducing SDS sample buffer; 2, Non-reducing sample buffer. **(D)** PreScission-cleaved YFP-S_RBD. 1, S_RBD from Ni-NTA IMAC; 2, S_RBD from SEC; 3, YFP washed off the Ni-NTA resin after on-bead cleavage, after removal of the protease. **(E)** Analytical SEC profile (280 nm absorbance) of S_RBD. Based on a calibration curve using the bovine γ-globulin (158 kDa), chicken ovalbumin (44 kDa) and horse myoglobin (17 kDa) peaks, the retention volume suggests an approximate molecular mass of 37 kDa. The calculated molecular weight of S_RBD, based on protein sequence, excluding glycosylations, is 25 kDa. The SDS-PAGE (shown in **D**) indicates a size of ∼35 kDa for the glycosylated S_RBD.

Based on analysis by SDS-PAGE, the protein purity was already high after Ni-NTA IMAC. The SEC profile of the YFP-S_RBD fusion protein confirmed the high purity and also showed that the protein solution was monodisperse. The negative staining electron micrograph confirms that the protein solution is monodisperse and individual particles are well distributed (Figure 4A).

Incubation of the purified fusion protein with the enzyme PNGase F resulted in slightly faster migration on SDS-PAGE, confirming the presence of N-glycosylations in the expressed protein (Figure 3A). Mass spectrometry analysis confirmed the presence of glycosylations and a reduction thereof upon PNGase treatment (Figure 4B). The fusion protein migrated slightly faster on SDS-PAGE in non-reducing conditions than in reducing conditions, indicating the presence of disulfide bonds in the protein domain (Figure 4C).

To test whether the S_RBD protein retains its properties after removal of the fluorescent protein tag, the YFP was removed by rhinovirus 3C protease cleavage. Four hundred microliters Ni-NTA resin were loaded with protein in five steps with a total of ∼260 ml expression medium. After washing, the Ni-NTA resin was incubated overnight in the presence of PreScission protease (GST-tagged human rhinovirus 3C protease). The YFP was then washed off and collected, while the His-tagged S_RBD protein remained on the column. The protein was eluted from the now colorless Ni-NTA resin using an imidazole-containing buffer and analyzed by SDS-PAGE (Figure 4D). The collected cleaved-off YFP was also analyzed on SDS-PAGE, after incubation with Glutathione sepharose 4B to remove the GST-tagged protease (Figure 4D).

The hS_RBD protein was analyzed by analytical SEC. There was a single main peak at approximately the expected retention volume, with only a slight shoulder, confirming that the S_RBD domain retained its solubility and monodispersity after removal of the YFP fusion protein (Figure 4E).

**FIGURE 5:**
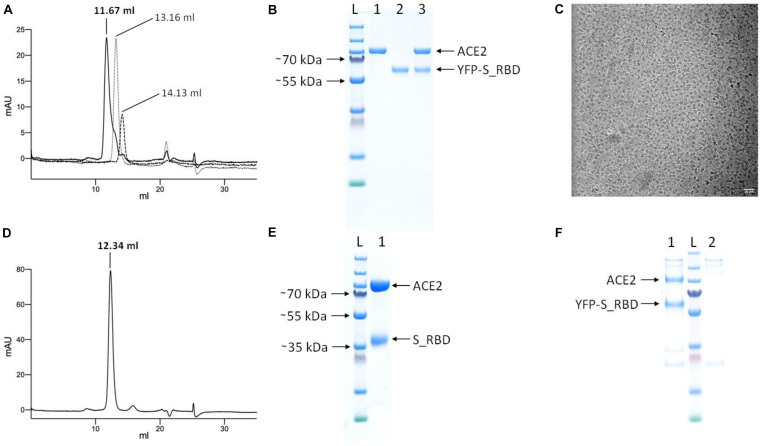
Analysis of complex formation by analytical SEC and pull-down assays. **(A)** Overlay of the 280 nm absorbance SEC traces from the complex (YFP-S_RBD/ACE2 run together, solid black line), ACE2 run separately (fine dotted line), and YFP-hS_RBD run separately (coarse dotted line). **(B)** SDS-PAGE analysis of the proteins. L, Ladder; 1, ACE2; 2; YFP-S_RBD; 3, complex eluted from SEC. **(C)** Cryo-EM micrograph of the ACE2/YFP-S_RBD complex Scale bar 20 nm. **(D)** 280 nm absorbance SEC trace of the complex of ACE2 with S_RBD (after proteolytic removal of YFP). **(E)** SDS-PAGE analysis of the complex eluted from **(D)**. Based on a calibration curve using the bovine γ-globulin (158 kDa), chicken ovalbumin (44 kDa) and horse myoglobin (17 kDa) peaks, the retention volume suggests the following approximate molecular masses: YFP-S_RBD-ACE2 complex: 168 kDa, ACE2 peptidase domain *=* 121 kDa, YFP-S_RBD *=* 90 kDa. Cleaved complex (S_RBD-ACE2) *=* 147 kDa. **(F)** Pull-down assay using Anti-FLAG affinity resin, confirming the ACE2 peptidase domain—YFP-S_RBD interaction. The YFP-S_RBD construct contains a FLAG-tag downstream of the YFP, the ACE2 construct does not contain a FLAG-tag. 1, YFP-S_RBD—ACE2 complex purified via Anti-FLAG resin; L, Ladder; 2, control not containing YFP-S_RBD.

To test whether the purified YFP-S_RBD fusion protein binds its target receptor ACE2, we produced and purified human ACE2 peptidase domain and analyzed the separate proteins as well as the complex of the two proteins by analytical SEC experiments. The complex co-eluted in a peak at a reduced retention volume compared to the peak from ACE2 run alone or the peak of YFP-S_RDB run alone, clearly confirming complex formation (Figures 5A,B).

The cryo-EM micrograph (Figure 5C) shows that the protein complex meets the quality standard for structural biology, indicating good contrast and particle distribution.

To test whether the S_RBD domain retains its ACE2-binding activity after proteolytic removal of the YFP, the analytical SEC experiment was repeated with PreScission protease cleaved, purified S_RBD. The two proteins co-eluted in a peak at a reduced retention volume compared to the separate proteins, confirming binding (Figures 5D,E).

The YFP-S_RBD—ACE2 peptidase interaction was further confirmed by a pull-down assay using Anti-FLAG affinity resin (Figure 5F).

## Discussion

Due to the ongoing COVID-19 pandemic, there is a very large demand for high-quality 2019-nCoV proteins for a wide range of research purposes, also by laboratories that only recently started COVID-related research. Here, we describe a detailed protocol for the production of the receptor-binding domain of the nCoV-19 spike protein that only requires standard cell culture equipment and skills. Adherent cell cultures are maintained in cell culture flasks, producing a continuous supply of protein that can be purified from serum-free medium.

The RBD of the spike protein is the domain that directly interacts with the human receptor ACE2 (Yan et al., 2020). It is therefore one of the key protein domains in studies that address the recognition of cells by the virus and in studies related to the development of novel vaccines.

A very important feature of our fusion protein is that it contains a yellow fluorescent protein, which makes YFP-S_RBD useful for direct detection of putative viral docking sites on cells. Furthermore, in combination with an interacting protein (e.g., ACE2) that is labeled with a compatible fluorophore, YFP-S_RBD is suited for binding studies involving fluorescent spectroscopy methods such as Förster resonance energy transfer (FRET) or fluorescence cross-correlation spectroscopy (FCCS). Since no adequate antiviral therapy against COVID-19 is available to date, there are worldwide efforts to develop or repurpose drugs. The spike RBD represents one of the most promising targets for prophylactic protection and treatment of early infection (Cao et al., 2020; Riva et al., 2020; Su et al., 2020). In this context, the fusion protein would also be suited for drug discovery projects, for example for high-throughput fluorescence-based binding assays.

YFP-S_RBD furthermore provides a solid basis for the design of novel fusion proteins. For example, one of our future aims is to produce protein nanoparticles (Collu et al., 2020) that display multiple copies of the S_RBD epitope on their surface, as tool compounds for the development of novel antibody detection assays or as a strategy to design improved samples for vaccination against 2019-nCoV or future emerging diseases. Considering the high expression and secretion levels of YFP-S_RBD with an N-terminal IFNα2 signal peptide, it should be feasible to replace the YFP by a protein that forms oligomers. The use of self-assembling protein nanoparticles with an optimized display of viral epitopes has enabled important advances in vaccinology (Rappuoli and Serruto, 2019).

S_RBD comprises co-translational modifications (disulfide bonds and glycosylations) that are important for correct folding. For this reason, the protein is typically produced by secretion from eukaryotic cells (Wang et al., 2020; Wrapp et al., 2020). Only few fusion proteins (the Fc domain of IgG and human serum albumin) are routinely used in the context of secreted proteins (Dalton and Barton, 2014). Not all proteins are suited for secretion. Intracellular proteins can contain glycosylation motifs. If such proteins are guided to the secretory pathway via a signal peptide, glycosylation can interfere with correct protein folding.

Our aim was to use YFP, a yellow variant of GFP, as the fusion protein. YFP is routinely used in our lab for the biophysical characterization of proteins, for example by fluorescence-detection size-exclusion chromatography (Kawate and Gouaux, 2006), or as a component in fusion proteins containing limited flexibility for structural biology applications (e.g., PDB entry 6HR1, Collu et al., 2020). Analysis of the protein sequence of YFP revealed only one putative glycosylation site, located near the protein surface, suggesting that secretion should be feasible. Secretion of GFP has previously been achieved (Román et al., 2016), and GFP contains the same putative O-glycosylation site as YFP.

In our construct for ACE2 expression, which does not contain a fluorescent protein, we used the IL-2 signal peptide, which is commonly used for secretion (e.g., Yao et al., 2016).

The choice for using the interferon alpha 2 (IFNα2) signal peptide for our YFP-S_RBD fusion protein was based on reference (Román et al., 2016), which suggests improved secretion levels with this signal peptide.

## Data Availability Statement

The original contributions presented in the study are included in the article/Supplementary Materials, further inquiries can be directed to the corresponding author/s.

## Author Contributions

RB initiated and coordinated the project. TB sub-cloned the ACE2 construct. RB, TB, and GC performed the cell culture experiments, protein purification, and biochemical analysis. EP and TB carried out the electron microscopy experiments. AB performed the mass spectrometry analysis. TB carried out the pull-down assays. RB and TB wrote the manuscript with contributions from all authors. All authors contributed to the article and approved the submitted version.

## Conflict of Interest

The authors declare that the research was conducted in the absence of any commercial or financial relationships that could be construed as a potential conflict of interest.
