# Supplementary material for: Design, Expression, Purification, and Characterization of a YFP-Tagged 2019-nCoV Spike Receptor-Binding Domain Construct
Source: Front Bioeng Biotechnol. 2020 Dec 21;8:618615. doi: 10.3389/fbioe.2020.618615 (PMC7779597; doi:10.3389/fbioe.2020.618615)
Supplement: Supplementary file 1 [file Data_Sheet_1.pdf]

# Supplementary Information

## Sequences of the expression constructs

### YFP_S-RBD:

**DNA sequence of the expression region, which was cloned into the HindIII and XbaI sites of pcDNA 4/TO:**

AAGCTTAAAGAAGCGGCCGCC**ATG**GCTCTGACATTTGCTCTGCTGGTGGCTCTGCTGGTGCTGAGCTGCAAGAGCAGCTGTAG
CGTGGGCGGATCCGTCTCCAAGGGCGAGGAACTGTTTACCGGAGTGGTGCCCATTCTGGTGGAACTGGACGGAGACGTGAATG
GACACAAGTTCAGCGTGAGCGGAGAAGGCGAAGGCGACGCCACCTACGGAAAGCTGACACTGAAGTTCATTTGCACAACCGGC
AAGCTGCCCGTCCCTTGGCCCACCCTCGTGACAACATTCGGATACGGCCTCCAGTGTTTCGCTAGGTATCCCGACCACATGAA
GCAGCACGACTTTTTCAAGAGCGCCATGCCCGAAGGCTACGTCCAAGAGAGGACAATCTTCTTCAAGGATGACGGCAACTATA
AGACAAGAGCCGAGGTCAAGTTCGAGGGAGACACACTGGTGAATAGAATCGAACTCAAGGGCATCGACTTCAAGGAGGATGGC
AATATTCTGGGCCACAAGCTGGAGTACAATTACAACAGCCACAACGTGTACATCATGGCCGACAAACAGAAGAACGGCATCAA
GGTGAACTTCAAGATTAGACACAACATCGAGGACGGCTCCGTGCAACTGGCCGACCACTATCAGCAGAATACCCCTATCGGCG
ATGGACCCGTGCTGCTGCCCGACAACCACTATCTGTCCTACCAGAGCGCTCTGAGCAAGGACCCCAACGAGAAGAGGGATCAC
ATGGTGCTGCTGGAGTTCGTGACCGCCGCCGGCATCACACTGGGCATGGACGAGCTGTACAAGGGCAGCGGCAGCGACTACAA
AGACGACGACGACAAAGGCTCTCTCGAGGTGCTGTTCCAAGGCCCCAGAGTCCAGCCCACCGAAAGCATCGTGAGGTTCCCCA
ACATCACCAACCTCTGCCCCTTCGGCGAAGTGTTTAATGCCACCAGATTCGCCAGCGTGTACGCTTGGAATAGAAAGAGGATC
TCCAACTGTGTGGCCGACTATAGCGTGCTCTACAACAGCGCCAGCTTCAGCACCTTCAAATGCTACGGAGTGTCCCCCACCAA
ACTGAACGATCTGTGCTTCACCAACGTGTACGCTGACTCCTTCGTGATTAGGGGCGACGAAGTGAGACAAATCGCTCCCGGAC
AGACCGGCAAGATCGCCGACTACAACTACAAGCTGCCCGACGACTTTACCGGCTGTGTGATCGCTTGGAACAGCAACAATCTC
GACTCCAAGGTCGGCGGCAACTACAATTATCTGTATAGACTGTTTAGAAAGTCCAATCTGAAGCCTTTCGAGAGGGACATCAG
CACCGAGATCTATCAAGCCGGCTCCACCCCTTGTAATGGCGTGGAGGGCTTCAACTGCTATTTCCCTCTGCAGAGCTACGGCT
TCCAGCCTACAAACGGCGTGGGATACCAGCCCTATAGGGTCGTGGTGCTGAGCTTTGAGCTGCTGCATGCTCCCGCCACCGTG
TGCGGCCCCCACCATCATCACCACCATCACCACTGATAATCTAGA

**Protein sequence (signal peptide, YFP, HRV3C protease site, S_RBD):**

MALTFALLVALLVLSCKSSCSVGGSVSKGEELFTGVVPILVELDGDVNGHKFSVSGEGEGDATYGKLTLKFICTTGKLPVPWP
TLVTTFGYGLQCFARYPDHMKQHDFFKSAMPEGYVQERTIFFKDDGNYKTRAEVKFEGDTLVNRIELKGIDFKEDGNILGHKL
EYNYNSHNVYIMADKQKNGIKVNFKIRHNIEDGSVQLADHYQQNTPIGDGPVLLPDNHYLSYQSALSKDPNEKRDHMVLLEFV
TAAGITLGMDELYKGSGSDYKDDDDKGSLEVLFQGPRVQPTESIVRFPNITNLCPFGEVFNATRFASVYAWNRKRISNCVADY
SVLYNSASFSTFKCYGVSPTKLNDLCFTNVYADSFVIRGDEVRQIAPGQTGKIADYNYKLPDDFTGCVIAWNSNNLDSKVGGN
YNYLYRLFRKSNLKPFERDISTEIYQAGSTPCNGVEGFNCYFPLQSYGFQPTNGVGYQPYRVVVLSFELLHAPATVCGPHHHH
HHHH**

### ACE2 peptidase domain:

**DNA sequence of the expression region, which was cloned into the KpnI and NotI sites of pcDNA 4/TO:**

GGTACCGCCACC**ATG**TATAGAATGCAGCTGCTCAGCTGCATCGCTCTGTCTCTGGCTCTGGTCACAAACAGCAGCACCATTGA
GGAGCAAGCCAAGACCTTTCTGGACAAATTTAACCACGAGGCCGAGGACCTCTTCTATCAAAGCTCTCTGGCCAGCTGGAACT
ACAATACAAACATTACCGAGGAGAACGTGCAGAACATGAACAACGCCGGCGATAAGTGGAGCGCCTTTCTGAAGGAGCAGAGC
ACACTGGCCCAAATGTACCCCCTCCAAGAGATTCAGAATCTGACCGTGAAGCTGCAGCTGCAAGCTCTGCAGCAGAACGGAAG
CTCCGTGCTCAGCGAGGATAAGAGCAAGAGACTCAACACCATTCTGAACACAATGTCCACCATCTACTCCACCGGAAAGGTCT
GCAACCCCGACAATCCCCAAGAGTGTCTGCTGCTGGAGCCCGGACTGAACGAGATCATGGCCAACTCTCTGGACTACAACGAA
AGGCTGTGGGCTTGGGAGAGCTGGAGATCCGAGGTGGGCAAGCAGCTCAGACCTCTGTACGAGGAGTACGTGGTGCTGAAGAA
CGAGATGGCTAGAGCTAACCACTACGAGGACTATGGCGACTATTGGAGGGGCGATTACGAAGTGAACGGCGTCGACGGATATG
ACTATTCTAGAGGCCAGCTGATCGAAGACGTCGAGCACACCTTCGAAGAGATCAAACCTCTGTACGAGCATCTGCACGCTTAT
GTGAGAGCCAAGCTGATGAACGCCTACCCCTCCTACATTTCCCCCATTGGCTGTCTGCCCGCCCATCTGCTGGGCGATATGTG
GGGAAGATTCTGGACCAATCTGTACTCTCTGACCGTGCCTTTCGGCCAAAAGCCCAACATCGACGTCACCGATGCTATGGTCG
ATCAAGCATGGGATGCTCAGAGAATCTTCAAGGAGGCCGAGAAGTTCTTCGTCAGCGTGGGACTGCCCAACATGACCCAAGGC
TTCTGGGAGAATAGCATGCTCACAGACCCCGGCAACGTGCAGAAGGCTGTGTGCCACCCTACCGCTTGGGATCTGGGCAAAGG
CGACTTTAGGATTCTGATGTGCACCAAGGTGACAATGGACGATTTTCTGACCGCCCACCACGAAATGGGCCACATCCAATACG
ACATGGCCTACGCCGCTCAGCCCTTTCTGCTGAGAAACGGAGCCAACGAGGGCTTTCACGAGGCTGTGGGCGAGATCATGTCT
CTGAGCGCCGCTACCCCCAAGCATCTGAAGAGCATCGGACTGCTGTCCCCCGACTTCCAAGAGGATAACGAGACCGAGATCAA
CTTTCTGCTGAAGCAAGCTCTGACCATCGTGGGCACACTGCCCTTCACCTACATGCTGGAGAAATGGAGGTGGATGGTGTTCA
AGGGCGAGATCCCTAAGGATCAGTGGATGAAGAAGTGGTGGGAGATGAAGAGGGAAATCGTGGGCGTGGTGGAACCCGTGCCT
CACGACGAGACCTACTGCGATCCCGCCAGCCTCTTCCACGTGAGCAACGATTACTCCTTCATTAGATACTACACAAGAACACT
GTACCAGTTCCAATTTCAAGAGGCTCTGTGCCAAGCCGCCAAACATGAGGGACCTCTGCACAAGTGCGACATCAGCAACAGCA
CCGAAGCTGGCCAGAAGCTGTTCAACATGCTGAGGCTGGGAAAGAGCGAGCCTTGGACACTGGCTCTGGAAAACGTCGTGGGC
GCCAAGAACATGAACGTGAGGCCTCTGCTGAACTACTTTGAACCTCTGTTCACATGGCTCAAGGACCAGAACAAGAACTCCTT

CGTGGGATGGTCCACCGATTGGAGCCCCTACGCCGATCTGGAAGTGCTCTTCCAAGGCCCTCACCATCATCACCACCATCACC
ATTGATGAGCGGCCGC

**Protein sequence:**
MYRMQLLSCIALSLALVTNSSTIEEQAKTFLDKFNHEAEDLFYQSSLASWNYNTNITEENVQNMNNAGDKWSAFLKEQSTLAQ
MYPLQEIQNLTVKLQLQALQQNGSSVLSEDKSKRLNTILNTMSTIYSTGKVCNPDNPQECLLLEPGLNEIMANSLDYNERLWA
WESWRSEVGKQLRPLYEEYVVLKNEMARANHYEDYGDYWRGDYEVNGVDGYDYSRGQLIEDVEHTFEEIKPLYEHLHAYVRAK
LMNAYPSYISPIGCLPAHLLGDMWGRFWTNLYSLTVPFGQKPNIDVTDAMVDQAWDAQRIFKEAEKFFVSVGLPNMTQGFWEN
SMLTDPGNVQKAVCHPTAWDLGKGDFRILMCTKVTMDDFLTAHHEMGHIQYDMAYAAQPFLLRNGANEGFHEAVGEIMSLSAA
TPKHLKSIGLLSPDFQEDNETEINFLLKQALTIVGTLPFTYMLEKWRWMVFKGEIPKDQWMKKWWEMKREIVGVVEPVPHDET
YCDPASLFHVSNDYSFIRYYTRTLYQFQFQEALCQAAKHEGPLHKCDISNSTEAGQKLFNMLRLGKSEPWTLALENVVGAKNM
NVRPLLNYFEPLFTWLKDQNKNSFVGWSTDWSPYADLEVLFQGPHHHHHHHH**
